# Lipid-dependent Akt-ivity: where, when, and how

**DOI:** 10.1042/BST20190013

**Published:** 2019-05-30

**Authors:** Katharina M. Siess, Thomas A. Leonard

**Affiliations:** 1 Department of Structural and Computational Biology, Max F. Perutz Laboratories (MFPL), Campus Vienna Biocenter 5, 1030 Vienna, Austria; 2 Department of Medical Biochemistry, Medical University of Vienna, 1090 Vienna, Austria

**Keywords:** Akt, kinase, lipids, phosphorylation/dephosphorylation, PI(3, 4)P2, PI(3, 4, 5)P3

## Abstract

Akt is an essential protein kinase activated downstream of phosphoinositide 3-kinase and frequently hyperactivated in cancer. Canonically, Akt is activated by phosphoinositide-dependent kinase 1 and mechanistic target of rapamycin complex 2, which phosphorylate it on two regulatory residues in its kinase domain upon targeting of Akt to the plasma membrane by PI(3,4,5)P_3_. Recent evidence, however, has shown that, in addition to phosphorylation, Akt activity is allosterically coupled to the engagement of PI(3,4,5)P_3_ or PI(3,4)P_2_ in cellular membranes. Furthermore, the active membrane-bound conformation of Akt is protected from dephosphorylation, and Akt inactivation by phosphatases is rate-limited by its dissociation. Thus, Akt activity is restricted to membranes containing either PI(3,4,5)P_3_ or PI(3,4)P_2_. While PI(3,4,5)P_3_ has long been associated with signaling at the plasma membrane, PI(3,4)P_2_ is gaining increasing traction as a signaling lipid and has been implicated in controlling Akt activity throughout the endomembrane system. This has clear implications for the phosphorylation of both freely diffusible substrates and those localized to discrete subcellular compartments.

## Akt at a glance—the primary effector of PI3K signaling

**Figure 1.**
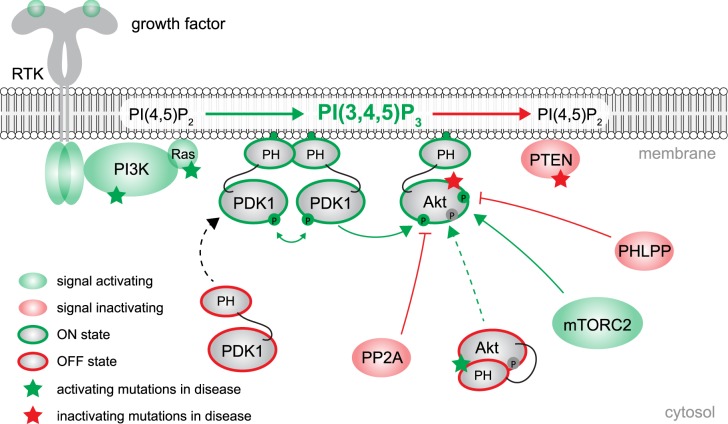
Akt at a glance – the primary effector of PI3K signaling. PI3K can be activated by the engagement of either RTKs or GPCRs with growth factors or hormones, respectively. PI(3,4,5)P_3_ production in the plasma membrane drives the translocation of PDK1 and Akt via their PH domains. PDK1 activates itself by homodimerization and trans-autophosphorylation, thereby permitting the activation of Akt by activation loop phosphorylation, aided by the regulatory phosphorylation of Akt by mTORC2. Akt is inactivated by PI(3,4,5)P_3_ and PI(3,4)P_2_ turnover, membrane dissociation, and dephosphorylation by the phosphatases PP2A and PHLPP.

Akt is one of the primary effectors of phosphoinositide 3-kinase (PI3K) signaling, regulating myriad processes including growth, proliferation, metabolism, and cell survival. Stimulation of G-protein-coupled receptors (GPCRs) or receptor tyrosine kinases (RTKs) through binding of extracellular growth factors or hormones [1] on the cell surface promotes downstream activation of class I PI3K (Figure 1). PI3K catalyzes the phosphorylation of phosphatidylinositol-4,5-bisphosphate (PI(4,5)P_2_) to phosphatidylinositol-3,4,5-trisphosphate [PI(3,4,5)P_3_] [2], while phosphatidylinositol-3,4-bisphosphate [PI(3,4)P_2_] is produced through either dephosphorylation of PI(3,4,5)P_3_ by the lipid phosphatase SHIP at the plasma membrane [3,4], or by phosphorylation of phosphatidylinositol-4-phosphate [PI(4)P] by class II PI3K [5,6]. By virtue of its pleckstrin homology (PH) domain, which binds both PI(3,4,5)P_3_ and PI(3,4)P_2_[7–9], Akt can transduce the PI3K output signal into a downstream response.

**Figure 2.**
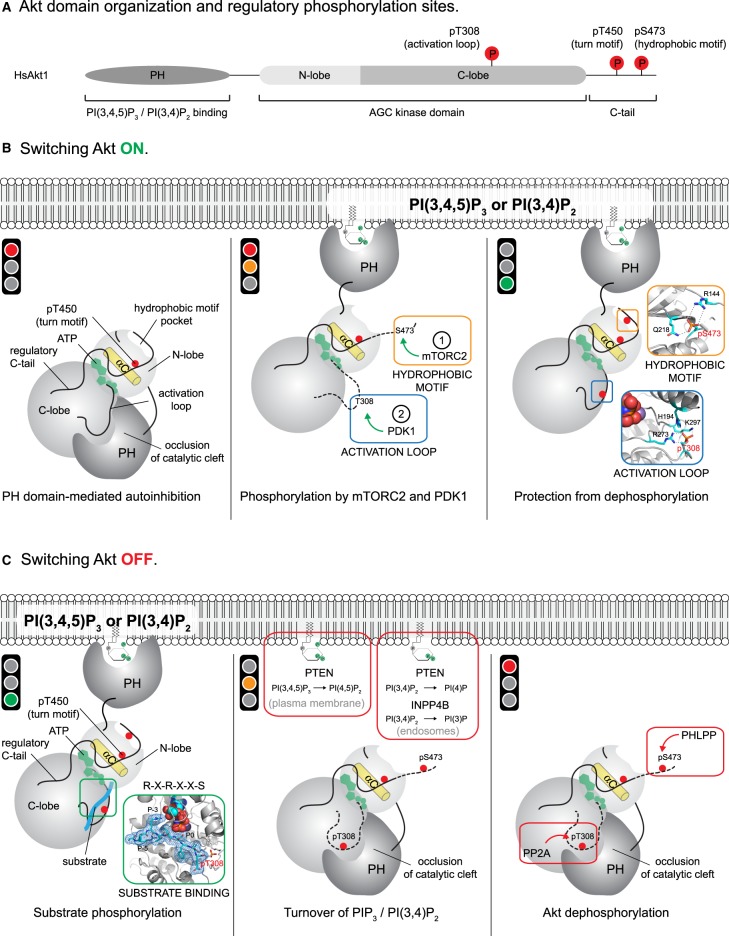
Switching Akt on and off. (**A**) Domain schematic of human Akt1. Regulatory phosphorylation sites are highlighted in red. (**B**) Switching Akt on. *Left panel* – Akt activity in the cytosol is blocked by its PH domain. Docking of the PH domain to the kinase domain occludes substrate binding and sequesters the activation loop and probably also the hydrophobic motif in a conformation inaccessible to PDK1 and mTORC2. *Middle panel* – PI(3,4,5)P_3_ generation in the plasma membrane leads to the binding of Akt and consequent displacement of its PH domain from its autoinhibited conformation, concomitantly exposing the activation loop and hydrophobic motif for phosphorylation. mTORC2 phosphorylation of the hydrophobic motif creates a docking site for PDK1, which subsequently mediates phosphorylation of the activation loop. *Right panel* – activation loop and hydrophobic motif phosphorylation drive a disorder-to-order transition of both segments, stabilized by ATP. The sequestration of both phosphorylated residues on the surface of the kinase domain protects them from dephosphorylation. (**C**) Switching Akt off. *Left panel* – Akt phosphorylated on both its activation loop and hydrophobic motif is primed for substrate binding and phosphorylation as long as Akt remains bound to PI(3,4,5)P_3_ or PI(3,4)P_2_. *Middle panel* – turnover of PI(3,4,5)P_3_ and PI(3,4)P_2_ by PTEN or PI(3,4)P_2_ by INPP4 results in Akt dissociation and inactivation by its PH domain. Docking of the PH domain to the kinase domain displaces the phosphorylated activation loop and hydrophobic motif, rendering them accessible for dephosphorylation. *Right panel* – Akt is dephosphorylated in its activation loop and hydrophobic motif. PP2A and PHLPP have been identified as the respective phosphatases, but further work is required to determine precisely where and how Akt is dephosphorylated.

There are three mammalian isoforms of Akt (Akt1, 2 and 3), which share a conserved domain arrangement of an N-terminal PH domain followed by a C-terminal serine/threonine kinase domain (Figure 2A). While not the focus of this article, the physiological and pathological roles of Akt isoforms have been reviewed in [10] and an emerging body of evidence also links signaling specificity to isoform-specific subcellular localization or isoform-specific substrates [11].

Dysregulation of PI3K/Akt signaling is associated with various pathologies including cancer, overgrowth disorders, and metabolic disease [2]. Oncogenic mutations in *PIK3CA*, the gene encoding the catalytic subunit of PI3K (p110α), are second only to Ras in frequency, both of which drive the overproduction of PI(3,4,5)P_3_ at the plasma membrane and result in an amplification of Akt signaling [12,13]. Mutations in the tumor suppressor phosphatase and tensin homolog (PTEN) are the third most common in human cancers [14]. Loss of PTEN, which terminates PI3K signaling by dephosphorylating the 3′-phosphoinositide products of PI3K, leads to an increase in PI(3,4,5)P_3_ at the membrane and hyperactivated Akt [15,16]. These gain- and loss-of-function mutations are also found in several overgrowth disorders [17,18], therefore highlighting the requirement for lipid signaling to be tightly regulated.

This article reviews our current knowledge about the fundamental mechanisms that control Akt activity in the cell. We focus primarily on recent advances in our understanding of how the lipid second messengers PI(3,4,5)P_3_ and PI(3,4)P_2_ regulate Akt activity and how these lipids are generated, distributed, and turned over in cells. In addition to its regulation by lipids, we discuss the activation of Akt by phosphorylation and its inactivation by dephosphorylation, and the mechanisms by which Akt is pathologically hyperactivated. Finally, we provide a perspective on the open questions that require further investigation.

## Switching Akt on

Akt is a serine/threonine kinase of the AGC family, the hallmark of which is the presence of a C-terminal regulatory tail containing one or more phosphorylation sites [19]. In the absence of a lipid stimulus, Akt is constitutively phosphorylated on T450, in its so-called turn motif, which is one of two phosphorylation sites in its C-terminal tail (Figure 2A). Turn motif phosphorylation stabilizes Akt and protects it from degradation by promoting the interaction of the tail with a conserved basic patch on the surface of the N-lobe of the kinase domain [20] (Figure 2B, left panel).

The generation of either PI(3,4,5)P_3_ or PI(3,4)P_2_ initiates translocation of Akt to the plasma membrane, leading to its phosphorylation on two residues in its activation loop (T308 in Akt1) and hydrophobic motif (S473 in Akt1), by phosphoinositide-dependent kinase 1 (PDK1) [21,22] and mechanistic target of rapamycin complex 2 (mTORC2) [23], respectively (Figure 2B, middle panel). PDK1 is co-recruited to the plasma membrane by virtue of its own PH domain, which, like Akt, binds to both PI(3,4,5)P_3_ and PI(3,4)P_2_[24] and drives PDK1 activation by homodimerization and trans-autophosphorylation [25,26]. mTORC2, on the other hand, is recruited to the plasma membrane by the PH domain of its obligate component Sin1 in a PI(3,4,5)P_3_-independent manner, leading to the suggestion that Akt hydrophobic motif phosphorylation is driven by the recruitment of Akt to constitutively active mTORC2 localized on distinct subcellular compartments [27].

Phosphorylation of the hydrophobic motif of the AGC kinases S6 kinase (S6K) and serum and glucocorticoid-regulated kinase 1 (SGK1), or a phosphomimetic at this site in atypical protein kinase C (aPKC) and protein kinase C-related kinase 2 (PRK2), enhances their activation by PDK1 [28,29], suggesting that hydrophobic motif phosphorylation by mTORC2 precedes activation loop phosphorylation by PDK1. While PDK1 itself lacks a hydrophobic motif, a hydrophobic pocket in the kinase domain of PDK1, called the PIF (PDK1-interacting fragment) pocket, binds to peptides containing a hydrophobic motif [30]. Phosphorylation of a conserved serine residue in the hydrophobic motif consensus sequence FXXFS or the presence of a phosphomimetic in place of the serine promotes this interaction [28,29], leading to the concept that hydrophobic motif phosphorylation of PDK1 substrates creates a docking site for PDK1 recruitment, thereby driving activation loop phosphorylation. While Akt was initially suggested to be activated by PDK1 independently of hydrophobic motif phosphorylation, the kinase domain of Akt bearing a phosphomimetic aspartate in its hydrophobic motif is a significantly better substrate for PDK1, and this is abrogated by mutation of the PIF pocket in PDK1 [28]. More recent work indicates that Akt bearing a phosphomimetic aspartate in its hydrophobic motif is also more efficiently phosphorylated by PDK1 in solution [31,32] and that disruption of the PIF pocket mechanism in Akt impacts Akt activation and sensitizes Akt to PDK1 inhibitors [33]. On balance, all indications are that activation loop phosphorylation of Akt by PDK1 depends on both its co-recruitment to PI(3,4,5)P_3_- or PI(3,4)P_2_-containing membranes and phosphorylation of its hydrophobic motif by mTORC2. Hydrophobic motif phosphorylation likely drives PDK1-mediated activation loop phosphorylation by both strengthening the interaction of PDK1 [32] with Akt and possibly positioning Akt optimally for catalysis.

In addition to enhancing activation loop phosphorylation by PDK1, phosphorylation of the hydrophobic motif promotes docking to a hydrophobic pocket in the kinase domain of Akt itself, thereby driving a disorder-to-order transition of the hydrophobic motif and the αC helix. This leads to the formation of a network of stabilizing interactions between the αC helix, ATP, and residues in the activation segment including pT308, which serve to immobilize the activation loop on the surface of the kinase domain [34,35]. However, precisely what triggers the dissociation of the phosphorylated hydrophobic motif of Akt from PDK1 and its subsequent intramolecular engagement is currently unknown. Activation loop phosphorylation by PDK1 may be a prerequisite for pS473 binding to the PIF pocket of Akt, in which case the intramolecular interaction may be favored post-activation loop phosphorylation, though this will need careful investigation. Nevertheless, dual phosphorylation of Akt effectively creates the substrate-binding cleft and organizes the catalytic residues essential for phospho-transfer [34] (Figure 2B, right panel; Figure 2C, left panel). A recent study suggests that S473 phosphorylation is stabilized by a basic patch in the PH–kinase domain linker containing R144 (Figure 2B, right panel, orange inset box), mutation of which results in a ∼50-fold reduction in catalytic efficiency [32]. Since the side chain of R144 cannot reach the side chain of an aspartate phosphomimetic, these observations raise the obvious question of whether D473 is a faithful mimic of pS473, despite its use in multiple cellular and *in vitro* studies.

That phosphorylation of Akt was required for its activity was recognized from the very beginning. However, early studies provided contradictory data on whether PI(3,4,5)P_3_ binding also contributed to an increase in Akt activity by relieving an autoinhibitory interaction between its PH and kinase domains. Some studies have hinted at such a conformation: Akt binding to PI(3,4,5)P_3_ enhances activation loop phosphorylation by PDK1 [22] and deletion of the PH domain of Akt promotes hydrophobic motif phosphorylation in an mTORC2-independent manner in Sin1 knockout fibroblasts [36]. Other studies including FRET and computational modeling [37–39], as well as the crystal structure of a C-terminally truncated Akt1 in complex with an allosteric inhibitor [40] and a follow-up mutational study [41] have strongly suggested the existence of an inactive conformation. However, it has only recently been demonstrated that Akt is directly activated *in vitro* by both PI(3,4,5)P_3_ and PI(3,4)P_2_ [42,43]. Mechanistically, lipid binding displaces the PH domain from the catalytic cleft, leading to an 8-fold increase in substrate binding. Mutation of two evolutionarily invariant, surface-exposed residues on the surface of the kinase domain, D323 and D325, uncouple kinase activity from PI(3,4,5)P_3_ and lead to Akt hyperphosphorylation, which is accompanied by a further 5-fold increase in affinity for the substrate. Displacement of the PH domain from the kinase domain results in enhanced membrane binding both *in vitro* and *in vivo*, indicating that the autoinhibitory interface also sequesters the PI(3,4,5)P_3_-binding pocket in an inaccessible conformation. Together, binding to PI(3,4,5)P_3_ and phosphorylation lead to a combined 40-fold increase in substrate binding. Consistent with these observations, Akt was only observed to form a complex with a model substrate *in vivo* when bound to PI(3,4,5)P_3_- or PI(3,4)P_2_-containing membranes [42].

Recent small-angle X-ray scattering (SAXS) and hydrogen–deuterium exchange mass spectrometry (HDX-MS) experiments have elucidated the conformational changes accompanying PI(3,4,5)P_3_ binding [43]. In the absence of PI(3,4,5)P_3_, phosphorylation of Akt is impeded, since both the activation loop and the hydrophobic motif are sequestered in the autoinhibited conformation. Binding of Akt to either PI(3,4,5)P_3_ or PI(3,4)P_2_ relieves autoinhibition by the PH domain by displacing it from the catalytic cleft and concomitantly liberating both the activation loop and hydrophobic motif for phosphorylation (Figure 2B, middle panel). Stoichiometric activation loop phosphorylation, at least in the context of a hydrophobic motif phosphomimetic (D473), is insufficient to overcome the dependency on lipid binding for full activation. In summary, Akt functions like a logic gate: both PI(3,4,5)P_3_/PI(3,4)P_2_ binding and phosphorylation are required to activate the kinase.

Docking of the phosphorylated activation loop and hydrophobic motif to the kinase domain has implications for the processivity of Akt signaling and its inactivation by phosphatases. Several studies have demonstrated that binding of ATP, but not ADP, protects Akt from dephosphorylation through caging of the phosphorylated activation loop and hydrophobic motif, which results in their restricted accessibility [44–46]. In other words, the same network of interactions that stabilize the active conformation of Akt protects it from dephosphorylation. Binding of ATP-competitive, but not allosteric inhibitors, also locks Akt in a hyperphosphorylated state and protects it from dephosphorylation [44,47]. Structural and mutational analyses in combination with molecular dynamics simulations have shown that the phosphorylation of T308 leads to formation of stabilizing contacts with R273 and H194, two residues in the nucleotide-binding pocket, which shield the regulatory residue from dephosphorylation [44,46]; mutation of either residue to alanine renders T308 susceptible to phosphatases [44]. Importantly, mutation of Q218 in the kinase domain, which forms a hydrogen bond with the phosphate group of pS473 (Figure 2B, right panel) also leads to decreased steady-state Akt phosphorylation [44]. In conclusion, phosphorylation of Akt results in a set of intramolecular interactions in which pT308 and pS473 co-operate to form the substrate-binding cleft and organize the catalytic machinery; as a consequence, they are protected from dephosphorylation in the presence of ATP. Since ATP concentrations in the cell are typically an order of magnitude greater than ADP, it seems likely that the ADP produced in a single catalytic cycle would be rapidly replaced with ATP. In this scenario, Akt would be permissive for iterative cycles of substrate phosphorylation, providing it remains in its active, membrane-bound conformation (Figure 2B, right panel; Figure 2C, left panel).

The ATP-dependent protection of phosphorylated Akt from dephosphorylation raises the obvious question of whether Akt requires PI(3,4,5)P_3_ or PI(3,4)P_2_ for activity once phosphorylated. While we have shown in two separate studies that phosphorylated Akt depends on PI(3,4,5)P_3_ or PI(3,4)P_2_ for full activity [42,43], a more recent study failed to observe the activation of Akt by PI(3,4,5)P_3_ [32]. Instead, the authors propose that phosphorylation of S473 in the hydrophobic motif promotes the disengagement of the PH domain from the kinase domain by facilitating an interaction with R144 in the PH–kinase linker. In this way, Akt could be activated by phosphorylation in the absence of PI(3,4,5)P_3_. However, there are significant problems with this model. First of all, the model relies on phosphorylation of the hydrophobic motif prior to PH domain disengagement when evidence suggests that these two events occur in the opposite order: deletion of the PH domain of Akt promotes hydrophobic motif phosphorylation in an mTORC2-independent manner [36], while PI(3,4,5)P_3_ binding results in displacement of the unphosphorylated hydrophobic motif from the kinase domain [43], suggesting that membrane binding elicits a conformational change that exposes the tail for phosphorylation. Secondly, the model implies that a single additional hydrogen bond made by pS473 to R144 is sufficient to disrupt an interface of ∼1500 Å^2^ buried surface area [40], which seems energetically unlikely.

So why do the authors of this study fail to observe Akt activation by PI(3,4,5)P_3_ or PI(3,4)P_2_? We previously demonstrated that the high concentration of magnesium (2–10 mM) routinely used in kinase assays inhibits Akt1 binding to PI(3,4,5)P_3_-containing liposomes [42]. When adjusted to levels that support ∼70% binding (0.2 mM), Akt is activated in a concentration-dependent manner by both PI(3,4,5)P_3_ and PI(3,4)P_2_, where the activity curve precisely mirrors the binding curve. The authors of the latest study dispute the influence of magnesium on PI(3,4,5)P_3_ binding [32], but their conclusion is based on analysis of the PH domain binding to soluble PI(3,4,5)P_3_ and not on the binding of full-length Akt1 to PI(3,4,5)P_3_-containing liposomes, which is more representative of physiological conditions. Furthermore, to activate Akt, the authors use a simple DOPC-PI(3,4,5)P_3_ liposome mixture for which they do not show evidence of Akt binding. At least in our hands, such a lipid composition does not support robust binding of full-length Akt1, which may explain the authors’ failure to observe activation by PI(3,4,5)P_3_.

In summary, the requirement for PI(3,4,5)P_3_ or PI(3,4)P_2_ for Akt activity is supported *in vitro* by biochemistry [42,43], mutagenesis [41–43], solution mapping of the autoinhibitory conformation [43], and a crystal structure in complex with an allosteric inhibitor [40]. *In vivo*, it is supported by diffusion measurements of the enzyme–substrate complex [42]. Nevertheless, the use of a phosphomimetic in the hydrophobic motif (D473) [43] is clearly only a proxy for S473 phosphorylation and further work will undoubtedly be required to unambiguously resolve the question of whether hydrophobic motif phosphorylation can activate Akt in a cellular context independently of either of these two lipids.

## Switching Akt off

The canonical pathway to Akt inactivation centers on the turnover of PI(3,4,5)P_3_ and PI(3,4)P_2_ by the lipid phosphatases PTEN and inositol polyphosphate 4-phosphatase (INPP4), respectively (Figure 2C, middle panel). PTEN hydrolyzes the 3′-phosphate of PI(3,4,5)P_3_ at the plasma membrane (reviewed in [15,16]), while INPP4 catalyzes the dephosphorylation of PI(3,4)P_2_ to PI(3)P at the plasma membrane and on endomembranes [48,49]. Turnover of PI(3,4,5)P_3_ and PI(3,4)P_2_ limits the activation of both Akt and PDK1 by phosphorylation. Evidence also indicates that it limits Akt activity by returning Akt to its autoinhibited conformation in which its substrate-binding cleft is blocked by its PH domain [43] (Figure 2C, middle panel).

**Figure 3.**
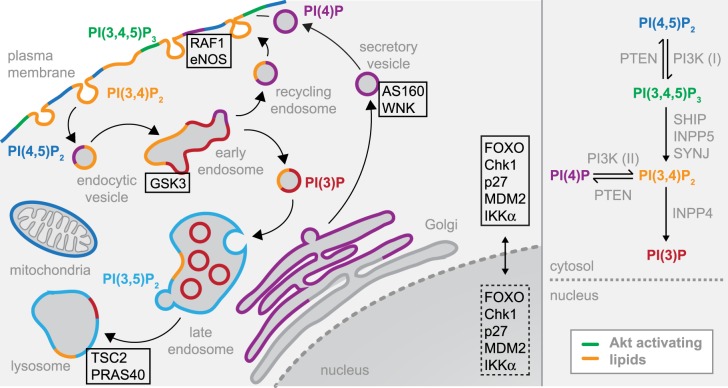
Spatiotemporal dynamics of PI(3,4,5)P_3_ and PI(3,4)P_2_. *Left panel* – Distribution of signaling phosphoinositides in the endomembranes of cells. PI(3,4,5)P_3_- and PI(3,4)P_2_-containing membranes are indicated in green and orange, respectively. Potential sites of Akt activity are expected to coincide with these lipids. Bona fide Akt substrates [50] are indicated in the black boxes at specific subcellular compartments. Substrates that exert their function in the nucleus, but are phosphorylated by Akt in the cytoplasm, are indicated with a dashed black box. *Right panel* – inter-conversion of phosphoinositides and the enzymes responsible.

We recently demonstrated that stoichiometric activation loop phosphorylation in combination with a hydrophobic motif phosphomimetic (D473) is insufficient to override the dependency on PI(3,4,5)P_3_ or PI(3,4)P_2_ for full activity [43]. HDX-MS analysis of this species in the absence of PI(3,4,5)P_3_ indicates that the autoinhibitory interface between the PH and kinase domains is maintained, but that the phosphorylated activation loop is considerably more labile than its unphosphorylated counterpart [43]. This prompted us to hypothesize that dissociation from the membrane triggers PH domain-mediated autoinhibition, which expels the activation loop from its docked conformation on the kinase domain (Figure 3, middle panel). To test whether dissociation could prime Akt for dephosphorylation, we examined the dephosphorylation kinetics of full-length Akt and its isolated kinase domain. Significantly, only the kinase domain is protected from dephosphorylation in the presence of ATP [43]. We concluded that the PH domain promotes Akt dephosphorylation. This provides a potential explanation for our earlier observations that Akt dephosphorylation in cells is rate-limited by its dissociation from the membrane [42]. Together, these findings suggest that Akt inactivation may actually occur in the cytosol, post-membrane dissociation.

Two phosphatases have been implicated in the dephosphorylation of Akt: protein phosphatase 2A (PP2A) and the PH domain leucine-rich repeat protein phosphatase (PHLPP) [51,52] (Figure 2C, right panel). The specificity of PP2A for its substrates is generally accomplished by incorporation of specific regulatory subunits into a heterotrimeric holoenzyme assembly [53]. PP2A specifically dephosphorylates Akt at T308 through incorporation of the regulatory subunit B55α [54], but we still lack a mechanistic understanding of T308-specific dephosphorylation. PHLPP has been proposed to selectively dephosphorylate S473 in the hydrophobic motif of Akt [51]. However, it is unclear how the specificity for S473 over T308 is achieved or why hydrophobic motif dephosphorylation *in vivo* would not affect T308 phosphorylation given their synergistic stabilization and similar dephosphorylation kinetics [44]. Furthermore, PHLPP is a membrane-associated phosphatase, either by directly binding to lipids via its PH domain [55] or indirectly through association of its PDZ ligand with membrane-resident scaffolding proteins [56,57]. Logically, this implies that substrate dephosphorylation happens at, or in close proximity to, the membrane. However, since Akt is protected from dephosphorylation when bound to PI(3,4,5)P_3_ and ATP [43], it raises the question of whether Akt is a substrate for dephosphorylation when membrane bound. The location and conditions under which Akt is dephosphorylated should, therefore, be an important focus of future studies.

## Spatiotemporal dynamics of PI(3,4,5)P_3_ and PI(3,4)P_2_

Accumulating evidence indicates that the key to both Akt activation and activity is PI(3,4,5)P_3_ or PI(3,4)P_2_. It therefore follows that subcellular Akt activity will be governed by the spatial and temporal distribution of these two lipids. In this section, we discuss where, when, and how these lipids are produced at different subcellular compartments, the enzymes that are responsible for both their production and turnover, and the implications for the activation of Akt and its substrate specificity.

In serum-starved cells, PI(3,4,5)P_3_ accounts for <0.1 mol% of all phospholipids, while growth factor stimulation transiently increases PI(3,4,5)P_3_ levels to ∼0.5 mol% [4]. Following growth factor binding, RTKs are rapidly internalized and degraded via the lysosomal pathway or recycled to the plasma membrane [58]. However, there is mounting evidence that signaling of the internalized receptors is actually sustained at endomembrane sites. PI(3,4)P_2_ itself has been found in endocytic vesicles [5,59], early endosomes [4,59], recycling endosomes [60], and late endosomes/lysosomes [61] (Figure 3), highlighting the existence of potential Akt signaling hubs in the cell interior. With respect to Akt activation, both PDK1 and mTORC2 have been found on endosomes [27,62]. Appl1, a protein involved in vesicle trafficking, Akt2, and its substrate GSK3β co-localize on endosomal membranes in a PI(3,4)P_2_-dependent manner [63], while another study indicates that class II PI3K-C2γ is responsible for sustained and isoform-specific Akt2 signaling on Rab5-positive early endosomes in glucose homeostasis [64]. Ratiometric imaging of PI(3,4,5)P_3_ and PI(3,4)P_2_ using high specificity fluorescence sensors has revealed that these lipids exhibit distinct spatiotemporal distributions in response to growth factor stimulation and that this leads to the differential regulation of Akt isoforms [4].

Previously believed to be just a byproduct of PI(3,4,5)P_3_ dephosphorylation, PI(3,4)P_2_ is increasingly being recognized as a signaling molecule in its own right, in addition to its known roles in membrane trafficking [5,59,65]. Indeed, Akt is activated *in vitro* to the same extent by PI(3,4,5)P_3_ and PI(3,4)P_2_ [43], which is consistent with the crystal structure of the PH domain of Akt1 in complex with inositol-1,3,4,5-tetrakisphosphate (IP_4_), in which the 5′-phosphate is not specifically recognized [9]. Soluble IP_4_, however, does not activate Akt *in vitro* [43]. These findings emphasize the importance of both the 3′-phosphate and the membrane environment as essential for Akt activity. Canonically, PI(3,4)P_2_ is generated by class II PI3K through 3′-phosphorylation of PI(4)P [6]. However, two recent studies have shown that the majority of endomembrane PI(3,4)P_2_ is actually produced through SHIP-mediated dephosphorylation of plasma membrane PI(3,4,5)P_3_ rather than by phosphorylation of PI(4)P [4,66]. Consistent with actively signaling pools of PI(3,4)P_2_ in the cell interior, targeting of PTEN to endomembranes has also been shown to convert PI(3,4)P_2_ to PI(4)P [67] in addition to its canonical role as a PI(3,4,5)P_3_ phosphatase.

Akt has been reported to phosphorylate more than 100 substrates, of which only a small fraction have been validated *in vitro* and *in vivo* [50]. Several of these substrates, including RAF1 and eNOS, localize to the plasma membrane. Other substrates, such as GSK3β localize to endosomes [63], while TSC2 and PRAS40, which is an inhibitory component of the mTORC1 complex [68,69], localize to the lysosome [70]. WNK and AS160 are found on both the plasma membrane and secretory vesicles [71,72] (Figure 3). While several substrates of Akt are found in the nucleus, which has led many to believe that Akt is active in the nucleus, evidence for nuclear PI(3,4,5)P_3_ or PI(3,4)P_2_ remains scarce [73]. Of these substrates, FOXO, Chk1, p27, MDM2, and IKKα shuttle between the nucleus and cytoplasm, and in principle could be phosphorylated in the cytoplasm [74,75]. In summary, the phosphorylation of a freely diffusible substrate is entirely consistent with membrane-restricted Akt activity, while the phosphorylation of a membrane-resident substrate will be dependent on the spatial and temporal distribution of PI(3,4,5)P_3_ or PI(3,4)P_2_. The local activation of Akt at subcellular compartments other than the plasma membrane will, of course, depend not only on the presence of PI(3,4,5)P_3_ or PI(3,4)P_2_ but also on the co-localization of PDK1 and mTORC2. While PDK1 binds to both PI(3,4,5)P_3_ and PI(3,4)P_2_ and therefore co-localizes with Akt, the mechanisms governing mTORC2 localization on endomembranes are less clear.

## Pathological hyperactivation and inactivation of Akt

The PI3K/Akt signaling pathway can be dysregulated at several different levels, and hot spot mutations in PI3K that up-regulate PI(3,4,5)P_3_ production or loss/inactivation of PTEN have already been extensively reviewed [2,12,16,76,77]. Importantly, however, several mutations in Akt itself have also been observed, which either disrupt the autoinhibitory interface between the PH and kinase domain and uncouple the enzyme from the lipid stimulus, lead to increased association of Akt with membranes, or inactivate Akt.

Akt was originally identified as a viral oncogene in which a sequence from the gag polyprotein was appended to the N-terminus of Akt [78]. Myristoylation of Akt leads to constitutive membrane association and Akt hyperactivation [79], presumably by increasing the on-rate of PI(3,4,5)P_3_ binding.

Mutation of a conserved glutamate in the PH domain (E17K) has been identified in breast cancer [80] as well as overgrowth disorders such as Proteus syndrome [81] and hemimegalencephaly [82]. E17K dramatically enhances the affinity of Akt for PI(4,5)P_2_ [83], thereby increasing association with the membrane in the absence of PI(3,4,5)P_3_ [80]. Although it was previously observed that this mutation also weakens the interaction between PH and kinase domains [41], it was more recently shown that its activity *in vitro* is still dependent on PI(3,4,5)P_3_ [42]. It remains to be determined whether E17K, in the context of full-length Akt, associates with the membrane at lower PI(3,4,5)P_3_ levels due to weakening of the autoinhibitory interface, but this seems a likely explanation for its hyperactivation and oncogenic potential.

From the dependency on PI(3,4,5)P_3_/PI(3,4)P_2_ for Akt activity, it logically follows that mutations in the autoinhibitory interface might uncouple Akt activity from the membrane. A screen of mutants in the PH–kinase interface, which was identified in the crystal structure of Akt1 in complex with an allosteric inhibitor [40], indicated many mutants that exhibited reduced PH–kinase domain association, resistance to allosteric inhibitors, and transforming activity *in vivo* [41]. Mutation of D323 and D325, surface-exposed and invariant residues on the kinase domain, leads to PI(3,4,5)P_3_-independent activity *in vitro*, hyperphosphorylation, and formation of a kinase–substrate complex in the cytosol of cells [42]. Biophysical and biochemical analysis has revealed an extended conformation, displacement of the PH domain from the catalytic cleft, and resistance to dephosphorylation in the presence of ATP [43]. Mutation of D323 in Akt1 has been observed in at least one case of urinary carcinoma [84] and the equivalent residue D322 in Akt3, the predominant isoform of Akt in the brain, in rare cases of extreme megalencephaly [85]. It should be noted that the pathological manifestation of disease depends on the nature of the mutation in Akt. Those that uncouple Akt activity from PI(3,4,5)P_3_ will undoubtedly have more severe consequences than those that simply enhance Akt activation by PI(3,4,5)P_3_, which may explain why they are comparatively rare in cancer [84].

While the majority of mutations lead to hyperactivation of Akt and increased signaling output, mutation of R274 to histidine in Akt2 inactivates the enzyme, leading to insulin resistance and diabetes [86]. Residing in the catalytic cleft of Akt, R274 is important for co-ordinating the phosphorylated T308 in the activation loop (Figure 2B, right panel). The shorter side chain of histidine cannot make the same stabilizing hydrogen bond, leading to Akt dephosphorylation and inactivation even in the presence of ATP [44].

**Perspectives**

- **Importance of the field:** Akt is a critical regulator of a diverse array of processes in the cell that co-ordinate growth, proliferation, and metabolism. Akt activity is up-regulated in a majority of human cancers that arise from aberrant PI3K signaling. Understanding the regulation of Akt by the products of PI3K is, therefore, of paramount importance in our efforts to rationalize both physiological and pathological signal transduction via this pathway.
- **Summary of current thinking:** The dependency of Akt on PI(3,4,5)P_3_ or PI(3,4)P_2_ for its activation has long been recognized, but whether it depends on either of these lipids for activity is still somewhat controversial. However, recent studies have clearly demonstrated that lipid binding relieves occlusion of the substrate-binding cleft by the PH domain and that activation loop phosphorylation is insufficient to overcome this requirement. Diffusion measurements of Akt inside cells have revealed the presence of a substantial pool of endomembrane-associated Akt consistent with recent studies demonstrating the presence of PI(3,4)P_2_ in endosomal compartments. Consistent with a requirement for PI(3,4,5)P_3_ or PI(3,4)P_2_, active Akt in the cell interior appears to be almost exclusively associated with a membrane compartment. There is currently little evidence for exclusively nuclear Akt substrates, nuclear pools of membrane-embedded PI(3,4,5)P_3_ or PI(3,4)P_2_, or active Akt in the nucleus.
- **Future directions:** With the exception of mTORC2 localization, Akt activation by PI(3,4,5)P_3_ or PI(3,4)P_2_ is now well understood. Future studies will help refine our understanding of how PI3K signaling is co-ordinated with mTORC2 phosphorylation of Akt; this is particularly important since hydrophobic motif phosphorylation appears to be a prerequisite for PDK1-mediated activation loop phosphorylation and is required for ATP-dependent stabilization of the active conformation on the membrane. Further studies are also required to unambiguously determine whether hydrophobic motif phosphorylation can activate Akt independently of lipids. The results of these studies will not only inform our understanding of Akt signaling but also of other AGC kinases, such as PKC and S6K, that depend on hydrophobic motif phosphorylation. Though the evidence strongly indicates that Akt activity is intrinsically coupled to engagement of PI(3,4,5)P_3_ or PI(3,4)P_2_ in membranes, the inactivation of Akt by phosphatases is comparatively poorly understood. We still have much to learn about precisely where, when, and how Akt is switched off.

## Abbreviations

GPCR: G-protein-coupled receptor
HDX-MS: hydrogen–deuterium exchange mass spectrometry
INPP4: inositol polyphosphate 4-phosphatase
IP_4_: inositol-1,3,4,5-tetrakisphosphate
mTORC2: mechanistic target of rapamycin complex 2
PDK1: phosphoinositide-dependent kinase 1
PH: pleckstrin homology
PHLPP: PH domain leucine-rich repeat protein phosphatase
PI3K: phosphoinositide 3-kinase
PKC: protein kinase C
PP2A: protein phosphatase 2A
PTEN: phosphatase and tensin homolog
RTK: receptor tyrosine kinase

## References

[1] Manning B.D. and Toker A. (2017) AKT/PKB signaling: navigating the network. Cell 169, 381–405 10.1016/j.cell.2017.04.001 28431241 PMC5546324

[2] Fruman D.A., Chiu H., Hopkins B.D., Bagrodia S., Cantley L.C. and Abraham R.T. (2017) The PI3K pathway in human disease. Cell 170, 605–635 10.1016/j.cell.2017.07.029 28802037 PMC5726441

[3] Bunney T.D. and Katan M. (2010) Phosphoinositide signalling in cancer: beyond PI3K and PTEN. Nat. Rev. Cancer 10, 342–352 10.1038/nrc2842 20414202

[4] Liu S.-L., Wang Z.-G., Hu Y., Xin Y., Singaram I., Gorai S. et al. (2018) Quantitative lipid imaging reveals a new signaling function of phosphatidylinositol-3,4-bisphosphate: isoform- and site-specific activation of Akt. Mol. Cell 71, 1092–1104.e5 10.1016/j.molcel.2018.07.035 30174291 PMC6214670

[5] Posor Y., Eichhorn-Gruenig M., Puchkov D., Schöneberg J., Ullrich A., Lampe A. et al. (2013) Spatiotemporal control of endocytosis by phosphatidylinositol-3,4-bisphosphate. Nature 499, 233–237 10.1038/nature12360 23823722

[6] Falasca M. and Maffucci T. (2012) Regulation and cellular functions of class II phosphoinositide 3-kinases. Biochem. J. 443, 587–601 10.1042/BJ20120008 22507127

[7] Frech M., Andjelkovic M., Ingley E., Reddy K.K., Falck J.R. and Hemmings B.A. (1997) High affinity binding of inositol phosphates and phosphoinositides to the pleckstrin homology domain of RAC/protein kinase B and their influence on kinase activity. J. Biol. Chem. 272, 8474–8481 10.1074/jbc.272.13.8474 9079675

[8] James S.R., Downes C.P., Gigg R., Grove S.J., Holmes A.B. and Alessi D.R. (1996) Specific binding of the Akt-1 protein kinase to phosphatidylinositol 3,4,5-trisphosphate without subsequent activation. Biochem. J. 315, 709–713 10.1042/bj3150709 8645147 PMC1217264

[9] Thomas C.C., Deak M., Alessi D.R. and van Aalten D.M.F. (2002) High-resolution structure of the pleckstrin homology domain of protein kinase B/Akt bound to phosphatidylinositol (3,4,5)-trisphosphate. Curr. Biol. 12, 1256–1262 10.1016/S0960-9822(02)00972-7 12176338

[10] Dummler B. and Hemmings B.A. (2007) Physiological roles of PKB/Akt isoforms in development and disease. Biochem. Soc. Trans. 35, 231–235 10.1042/BST0350231 17371246

[11] Toker A. and Marmiroli S. (2014) Signaling specificity in the Akt pathway in biology and disease. Adv. Biol. Regul. 55, 28–38 10.1016/j.jbior.2014.04.001 24794538 PMC4062840

[12] Thorpe L.M., Yuzugullu H. and Zhao J.J. (2015) PI3K in cancer: divergent roles of isoforms, modes of activation and therapeutic targeting. Nat. Rev. Cancer 15, 7–24 10.1038/nrc3860 25533673 PMC4384662

[13] Castellano E. and Downward J. (2011) RAS interaction with PI3K: more than just another effector pathway. Genes Cancer 2, 261–274 10.1177/1947601911408079 21779497 PMC3128635

[14] Lawrence M.S., Stojanov P., Mermel C.H., Robinson J.T., Garraway L.A., Golub T.R. et al. (2014) Discovery and saturation analysis of cancer genes across 21 tumour types. Nature 505, 495–501 10.1038/nature12912 24390350 PMC4048962

[15] Worby C.A. and Dixon J.E. (2014) PTEN. Annu. Rev. Biochem. 83, 641–669 10.1146/annurev-biochem-082411-113907 24905788

[16] Lee Y.-R., Chen M. and Pandolfi P.P. (2018) The functions and regulation of the PTEN tumour suppressor: new modes and prospects. Nat. Rev. Mol. Cell Biol. 19, 547–562 10.1038/s41580-018-0015-0 29858604

[17] Madsen R.R., Vanhaesebroeck B. and Semple R.K. (2018) Cancer-associated PIK3CA mutations in overgrowth disorders. Trends Mol. Med. 24, 856–870 10.1016/j.molmed.2018.08.003 30197175 PMC6185869

[18] Hobert J.A. and Eng C. (2009) PTEN hamartoma tumor syndrome: an overview. Genet. Med. 11, 687–694 10.1097/GIM.0b013e3181ac9aea 19668082

[19] Kannan N., Haste N., Taylor S.S. and Neuwald A.F. (2007) The hallmark of AGC kinase functional divergence is its C-terminal tail, a cis-acting regulatory module. Proc. Natl Acad. Sci. U.S.A. 104, 1272–1277 10.1073/pnas.0610251104 17227859 PMC1783090

[20] Facchinetti V., Ouyang W., Wei H., Soto N., Lazorchak A., Gould C. et al. (2008) The mammalian target of rapamycin complex 2 controls folding and stability of Akt and protein kinase C. EMBO J. 27, 1932–1943 10.1038/emboj.2008.120 18566586 PMC2486276

[21] Alessi D.R., James S.R., Downes C.P., Holmes A.B., Gaffney P.R., Reese C.B. et al. (1997) Characterization of a 3-phosphoinositide-dependent protein kinase which phosphorylates and activates protein kinase Bα. Curr. Biol. 7, 261–269 10.1016/S0960-9822(06)00122-9 9094314

[22] Stokoe D., Stephens L.R., Copeland T., Gaffney P.R., Reese C.B., Painter G.F. et al. (1997) Dual role of phosphatidylinositol-3,4,5-trisphosphate in the activation of protein kinase B. Science 277, 567–570 10.1126/science.277.5325.567 9228007

[23] Sarbassov D.D., Guertin D.A., Ali S.M. and Sabatini D.M. (2005) Phosphorylation and regulation of Akt/PKB by the rictor-mTOR complex. Science 307, 1098–1101 10.1126/science.1106148 15718470

[24] Currie R.A., Walker K.S., Gray A., Deak M., Casamayor A., Downes C.P. et al. (1999) Role of phosphatidylinositol 3,4,5-trisphosphate in regulating the activity and localization of 3-phosphoinositide-dependent protein kinase-1. Biochem. J. 337, 575–583 10.1042/bj3370575 9895304 PMC1220012

[25] Casamayor A., Morrice N.A. and Alessi D.R. (1999) Phosphorylation of Ser-241 is essential for the activity of 3-phosphoinositide-dependent protein kinase-1: identification of five sites of phosphorylation in vivo. Biochem. J. 342, 287–292 10.1042/bj3420287 10455013 PMC1220463

[26] Masters T.A., Calleja V., Armoogum D.A., Marsh R.J., Applebee C.J., Laguerre M. et al. (2010) Regulation of 3-phosphoinositide-dependent protein kinase 1 activity by homodimerization in live cells. Sci. Signal. 3, ra78 10.1126/scisignal.2000738 20978239

[27] Ebner M., Sinkovics B., Szczygieł M., Ribeiro D.W. and Yudushkin I. (2017) Localization of mTORC2 activity inside cells. J. Cell Biol. 216, 343–353 10.1083/jcb.201610060 28143890 PMC5294791

[28] Biondi R.M., Kieloch A., Currie R.A., Deak M. and Alessi D.R. (2001) The PIF-binding pocket in PDK1 is essential for activation of S6K and SGK, but not PKB. EMBO J. 20, 4380–4390 10.1093/emboj/20.16.4380 11500365 PMC125563

[29] Balendran A., Biondi R.M., Cheung P.C., Casamayor A., Deak M. and Alessi D.R. (2000) A 3-phosphoinositide-dependent protein kinase-1 (PDK1) docking site is required for the phosphorylation of protein kinase Cζ (PKCζ) and PKC-related kinase 2 by PDK1. J. Biol. Chem. 275, 20806–20813 10.1074/jbc.M000421200 10764742

[30] Balendran A., Casamayor A., Deak M., Paterson A., Gaffney P., Currie R. et al. (1999) PDK1 acquires PDK2 activity in the presence of a synthetic peptide derived from the carboxyl terminus of PRK2. Curr. Biol. 9, 393–404 10.1016/S0960-9822(99)80186-9 10226025

[31] Balzano D., Fawal M.-A., Velázquez J V., Santiveri C.M., Yang J., Pastor J. et al. (2015) Alternative activation mechanisms of protein kinase B trigger distinct downstream signaling responses. J. Biol. Chem. 290, 24975–24985 10.1074/jbc.M115.651570 26286748 PMC4599004

[32] Chu N., Salguero A.L., Liu A.Z., Chen Z., Dempsey D.R., Ficarro S.B. et al. (2018) Akt kinase activation mechanisms revealed using protein semisynthesis. Cell 174, 897–907.e14 10.1016/j.cell.2018.07.003 30078705 PMC6139374

[33] Najafov A., Shpiro N. and Alessi D.R. (2012) Akt is efficiently activated by PIF-pocket- and PtdIns(3,4,5)P_3_-dependent mechanisms leading to resistance to PDK1 inhibitors. Biochem. J. 448, 285–295 10.1042/BJ20121287 23030823

[34] Yang J., Cron P., Good V.M., Thompson V., Hemmings B.A. and Barford D. (2002) Crystal structure of an activated Akt/protein kinase B ternary complex with GSK3-peptide and AMP-PNP. Nat. Struct. Biol. 9, 940–944 10.1038/nsb870 12434148

[35] Yang J., Cron P., Thompson V., Good V.M., Hess D., Hemmings B.A. et al. (2002) Molecular mechanism for the regulation of protein kinase B/Akt by hydrophobic motif phosphorylation. Mol. Cell 9, 1227–1240 10.1016/S1097-2765(02)00550-6 12086620

[36] Warfel N.A., Niederst M. and Newton A.C. (2011) Disruption of the interface between the pleckstrin homology (PH) and kinase domains of Akt protein is sufficient for hydrophobic motif site phosphorylation in the absence of mTORC2. J. Biol. Chem. 286, 39122–39129 10.1074/jbc.M111.278747 21908613 PMC3234737

[37] Calleja V., Laguerre M., Parker P.J. and Larijani B. (2009) Role of a novel PH-kinase domain interface in PKB/Akt regulation: structural mechanism for allosteric inhibition. PLoS Biol. 7, e1000017 10.1371/journal.pbio.1000017 PMC2628406 19166270

[38] Calleja V., Alcor D., Laguerre M., Park J., Vojnovic B., Hemmings B.A. et al. (2007) Intramolecular and intermolecular interactions of protein kinase B define its activation in vivo. PLoS Biol. 5, e95 10.1371/journal.pbio.0050095 17407381 PMC1845162

[39] Calleja V., Laguerre M. and Larijani B. (2009) 3-D structure and dynamics of protein kinase B-new mechanism for the allosteric regulation of an AGC kinase. J. Chem. Biol. 2, 11–25 10.1007/s12154-009-0016-8 19568789 PMC2682354

[40] Wu W.-I., Voegtli W.C., Sturgis H.L., Dizon F.P., Vigers G.P.A. and Brandhuber B.J. (2010) Crystal structure of human AKT1 with an allosteric inhibitor reveals a new mode of kinase inhibition. PLoS ONE 5, e12913 10.1371/journal.pone.0012913 20886116 PMC2944833

[41] Parikh C., Janakiraman V., Wu W.-I., Foo C.K., Kljavin N.M., Chaudhuri S. et al. (2012) Disruption of PH-kinase domain interactions leads to oncogenic activation of AKT in human cancers. Proc. Natl Acad. Sci. U.S.A. 109, 19368–19373 10.1073/pnas.1204384109 23134728 PMC3511101

[42] Ebner M., Lučić I., Leonard T.A. and Yudushkin I. (2017) PI(3,4,5)P_3_ engagement restricts Akt activity to cellular membranes. Mol. Cell 65, 416–431.e6 10.1016/j.molcel.2016.12.028 28157504

[43] Lučić I., Rathinaswamy M.K., Truebestein L., Hamelin D.J., Burke J.E. and Leonard T.A. (2018) Conformational sampling of membranes by Akt controls its activation and inactivation. Proc. Natl Acad. Sci. U.S.A. 115, E3940–E3949 10.1073/pnas.1716109115 29632185 PMC5924885

[44] Chan T.O., Zhang J., Rodeck U., Pascal J.M., Armen R.S., Spring M. et al. (2011) Resistance of Akt kinases to dephosphorylation through ATP-dependent conformational plasticity. Proc. Natl Acad. Sci. U.S.A. 108, E1120–E1127 10.1073/pnas.1109879108 22031698 PMC3219155

[45] Lin K., Lin J., Wu W.-I., Ballard J., Lee B.B., Gloor S.L. et al. (2012) An ATP-site on-off switch that restricts phosphatase accessibility of Akt. Sci. Signal. 5, ra37 10.1126/scisignal.2002618 22569334

[46] Lu S., Deng R., Jiang H., Song H., Li S., Shen Q. et al. (2015) The mechanism of ATP-dependent allosteric protection of Akt kinase phosphorylation. Structure 23, 1725–1734 10.1016/j.str.2015.06.027 26256536 PMC7734571

[47] Okuzumi T., Fiedler D., Zhang C., Gray D.C., Aizenstein B., Hoffman R. et al. (2009) Inhibitor hijacking of Akt activation. Nat. Chem. Biol. 5, 484–493 10.1038/nchembio.183 19465931 PMC2783590

[48] Ivetac I., Munday A.D., Kisseleva M V., Zhang X.-M., Luff S., Tiganis T. et al. (2005) The type Iα inositol polyphosphate 4-phosphatase generates and terminates phosphoinositide 3-kinase signals on endosomes and the plasma membrane. Mol. Biol. Cell 16, 2218–2233 10.1091/mbc.e04-09-0799 15716355 PMC1087230

[49] Ivetac I., Gurung R., Hakim S., Horan K.A., Sheffield D.A., Binge L.C. et al. (2009) Regulation of PI(3)K/Akt signalling and cellular transformation by inositol polyphosphate 4-phosphatase-1. EMBO Rep. 10, 487–493 10.1038/embor.2009.28 19325558 PMC2680870

[50] Manning B.D. and Cantley L.C. (2007) AKT/PKB signaling: navigating downstream. Cell 129, 1261–1274 10.1016/j.cell.2007.06.009 17604717 PMC2756685

[51] Gao T., Furnari F. and Newton A.C. (2005) PHLPP: a phosphatase that directly dephosphorylates Akt, promotes apoptosis, and suppresses tumor growth. Mol. Cell 18, 13–24 10.1016/j.molcel.2005.03.008 15808505

[52] Brognard J., Sierecki E., Gao T. and Newton A.C. (2007) PHLPP and a second isoform, PHLPP2, differentially attenuate the amplitude of Akt signaling by regulating distinct Akt isoforms. Mol. Cell 25, 917–931 10.1016/j.molcel.2007.02.017 17386267

[53] Shi Y. (2009) Serine/threonine phosphatases: mechanism through structure. Cell 139, 468–484 10.1016/j.cell.2009.10.006 19879837

[54] Kuo Y.-C., Huang K.-Y., Yang C.-H., Yang Y.-S., Lee W.-Y. and Chiang C.-W. (2008) Regulation of phosphorylation of Thr-308 of Akt, cell proliferation, and survival by the B55alpha regulatory subunit targeting of the protein phosphatase 2A holoenzyme to Akt. J. Biol. Chem. 283, 1882–1892 10.1074/jbc.M709585200 18042541

[55] Park W.S., Do H.W., Whalen J.H., O'Rourke N.A., Bryan H.M., Meyer T. et al. (2008) Comprehensive identification of PIP3-regulated PH domains from *C. elegans* to *H. sapiens* by model prediction and live imaging. Mol. Cell 30, 381–392 10.1016/j.molcel.2008.04.008 18471983 PMC3523718

[56] Li X., Yang H., Liu J., Schmidt M.D. and Gao T. (2011) Scribble-mediated membrane targeting of PHLPP1 is required for its negative regulation of Akt. EMBO Rep. 12, 818–824 10.1038/embor.2011.106 21701506 PMC3147256

[57] Molina J.R., Agarwal N.K., Morales F.C., Hayashi Y., Aldape K.D., Cote G. et al. (2012) PTEN, NHERF1 and PHLPP form a tumor suppressor network that is disabled in glioblastoma. Oncogene 31, 1264–1274 10.1038/onc.2011.324 21804599 PMC3208076

[58] Goh L.K. and Sorkin A. (2013) Endocytosis of receptor tyrosine kinases. Cold Spring Harb. Perspect. Biol. 5, a017459 10.1101/cshperspect.a017459 23637288 PMC3632065

[59] He K., Marsland R. III, Upadhyayula S., Song E., Dang S., Capraro B.R. et al. (2017) Dynamics of phosphoinositide conversion in clathrin-mediated endocytic traffic. Nature 552, 410–414 10.1038/nature25146 29236694 PMC6263037

[60] Román-Fernández Á., Roignot J., Sandilands E., Nacke M., Mansour M.A., McGarry L. et al. (2018) The phospholipid PI(3,4)P_2_ is an apical identity determinant. Nat. Commun. 9, 5041 10.1038/s41467-018-07464-8 30487552 PMC6262019

[61] Marat A.L., Wallroth A., Lo W.-T., Müller R., Norata G.D., Falasca M. et al. (2017) mTORC1 activity repression by late endosomal phosphatidylinositol 3,4-bisphosphate. Science 356, 968–972 10.1126/science.aaf8310 28572395

[62] Jethwa N., Chung G.H.C., Lete M.G., Alonso A., Byrne R.D., Calleja V. et al. (2015) Endomembrane PtdIns(3,4,5)P_3_ activates the PI3K-Akt pathway. J. Cell Sci. 128, 3456–3465 10.1242/jcs.172775 26240177

[63] Schenck A., Goto-Silva L., Collinet C., Rhinn M., Giner A., Habermann B. et al. (2008) The endosomal protein Appl1 mediates Akt substrate specificity and cell survival in vertebrate development. Cell 133, 486–497 10.1016/j.cell.2008.02.044 18455989

[64] Braccini L., Ciraolo E., Campa C.C., Perino A., Longo D.L., Tibolla G. et al. (2015) PI3K-C2γ is a Rab5 effector selectively controlling endosomal Akt2 activation downstream of insulin signalling. Nat. Commun. 6, 7400 10.1038/ncomms8400 26100075 PMC4479417

[65] Wallroth A. and Haucke V. (2018) Phosphoinositide conversion in endocytosis and the endolysosomal system. J. Biol. Chem. 293, 1526–1535 10.1074/jbc.R117.000629 29282290 PMC5798284

[66] Goulden B.D., Pacheco J., Dull A., Zewe J.P., Deiters A. and Hammond GR V. (2019) A high-avidity biosensor reveals plasma membrane PI(3,4)P_2_ is predominantly a class I PI3K signaling product. J. Cell Biol. 218, 1066–1079 10.1083/jcb.201809026 30591513 PMC6400549

[67] Malek M., Kielkowska A., Chessa T., Anderson K.E., Barneda D., Pir P. et al. (2017) PTEN regulates PI(3,4)P_2_ signaling downstream of class I PI3K. Mol. Cell 68, 566–580.e10 10.1016/j.molcel.2017.09.024 29056325 PMC5678281

[68] Sancak Y., Thoreen C.C., Peterson T.R., Lindquist R.A., Kang S.A., Spooner E. et al. (2007) PRAS40 is an insulin-regulated inhibitor of the mTORC1 protein kinase. Mol. Cell 25, 903–915 10.1016/j.molcel.2007.03.003 17386266

[69] Haar E V., Lee S., Bandhakavi S., Griffin T.J. and Kim D.-H. (2007) Insulin signalling to mTOR mediated by the Akt/PKB substrate PRAS40. Nat. Cell Biol. 9, 316–323 10.1038/ncb1547 17277771

[70] Menon S., Dibble C.C., Talbott G., Hoxhaj G., Valvezan A.J., Takahashi H. et al. (2014) Spatial control of the TSC complex integrates insulin and nutrient regulation of mTORC1 at the lysosome. Cell 156, 771–785 10.1016/j.cell.2013.11.049 24529379 PMC4030681

[71] Lee B.-H., Min X., Heise C.J., Xu B.-E., Chen S., Shu H. et al. (2004) WNK1 phosphorylates synaptotagmin 2 and modulates its membrane binding. Mol. Cell 15, 741–751 10.1016/j.molcel.2004.07.018 15350218

[72] Gonzalez E. and McGraw T.E. (2009) Insulin-modulated Akt subcellular localization determines Akt isoform-specific signaling. Proc. Natl Acad. Sci. U.S.A. 106, 7004–7009 10.1073/pnas.0901933106 19372382 PMC2678468

[73] Ananthanarayanan B., Ni Q. and Zhang J. (2005) Signal propagation from membrane messengers to nuclear effectors revealed by reporters of phosphoinositide dynamics and Akt activity. Proc. Natl Acad. Sci. U.S.A. 102, 15081–15086 10.1073/pnas.0502889102 16214892 PMC1257695

[74] Agarwal A.K. (2018) How to explain the AKT phosphorylation of downstream targets in the wake of recent findings. Proc. Natl Acad. Sci. U.S.A. 115, E6099–E6100 10.1073/pnas.1808461115 29907611 PMC6142228

[75] Leonard T.A. (2018) Reply to Agarwal: activity against nuclear substrates is not necessarily mediated by nuclear Akt. Proc. Natl Acad. Sci. U.S.A. 115, E6101–E6102 10.1073/pnas.1808882115 29907610 PMC6142218

[76] Burke J.E. (2018) Structural basis for regulation of phosphoinositide kinases and their involvement in human disease. Mol. Cell 71, 653–673 10.1016/j.molcel.2018.08.005 30193094

[77] Hollander M.C., Blumenthal G.M. and Dennis P.A. (2011) PTEN loss in the continuum of common cancers, rare syndromes and mouse models. Nat. Rev. Cancer 11, 289–301 10.1038/nrc3037 21430697 PMC6946181

[78] Bellacosa A., Testa J.R., Staal S.P. and Tsichlis P.N. (1991) A retroviral oncogene, Akt, encoding a serine-threonine kinase containing an SH2-like region. Science 254, 274–277 10.1126/science.1833819 1833819

[79] Kohn A.D., Summers S.A., Birnbaum M.J. and Roth R.A. (1996) Expression of a constitutively active Akt Ser/Thr kinase in 3T3-L1 adipocytes stimulates glucose uptake and glucose transporter 4 translocation. J. Biol. Chem. 271, 31372–31378 10.1074/jbc.271.49.31372 8940145

[80] Carpten J.D., Faber A.L., Horn C., Donoho G.P., Briggs S.L., Robbins C.M. et al. (2007) A transforming mutation in the pleckstrin homology domain of AKT1 in cancer. Nature 448, 439–444 10.1038/nature05933 17611497

[81] Lindhurst M.J., Sapp J.C., Teer J.K., Johnston J.J., Finn E.M., Peters K. et al. (2011) A mosaic activating mutation in *AKT1* associated with the Proteus syndrome. N. Engl. J. Med. 365, 611–619 10.1056/NEJMoa1104017 21793738 PMC3170413

[82] Lee J.H., Huynh M., Silhavy J.L., Kim S., Dixon-Salazar T., Heiberg A. et al. (2012) De novo somatic mutations in components of the PI3K-AKT3-mTOR pathway cause hemimegalencephaly. Nat. Genet. 44, 941–945 10.1038/ng.2329 22729223 PMC4417942

[83] Landgraf K.E., Pilling C. and Falke J.J. (2008) Molecular mechanism of an oncogenic mutation that alters membrane targeting: Glu17Lys modifies the PIP lipid specificity of the AKT1 PH domain. Biochemistry 47, 12260–12269 10.1021/bi801683k 18954143 PMC2919500

[84] Forbes S.A., Beare D., Gunasekaran P., Leung K., Bindal N., Boutselakis H. et al. (2015) COSMIC: exploring the world's knowledge of somatic mutations in human cancer. Nucleic Acids Res. 43, D805–D811 10.1093/nar/gku1075 25355519 PMC4383913

[85] Alcantara D., Timms A.E., Gripp K., Baker L., Park K., Collins S. et al. (2017) Mutations of AKT3 are associated with a wide spectrum of developmental disorders including extreme megalencephaly. Brain 140, 2610–2622 10.1093/brain/awx203 28969385 PMC6080423

[86] George S., Rochford J.J., Wolfrum C., Gray S.L., Schinner S., Wilson J.C. et al. (2004) A family with severe insulin resistance and diabetes due to a mutation in AKT2. Science 304, 1325–1328 10.1126/science.1096706 15166380 PMC2258004

